# Agentic AI for trustworthy synthetic microbial genomics: a perspective on generation, validation, and governance

**DOI:** 10.3389/fbinf.2026.1903746

**Published:** 2026-08-12

**Authors:** Fahim Sufi

**Affiliations:** COEUS Institute, New Market, VA, United States

**Keywords:** agentic AI, biosecurity, genome scale benchmarking, genomic foundation models, metagenomics, reproducibility, synthetic data validation, synthetic microbial genomics

## Abstract

Synthetic microbial genomic data are becoming increasingly important for benchmarking microbial genome analysis pipelines, simulating rare taxa, evaluating metagenomic workflows, and supporting reproducible computational biology. Recent genomic foundation models demonstrate that biological sequences can be modelled at unprecedented scale, with emerging capacity for genome-level interpretation, generation, and design. However, the scientific value of synthetic microbial genomic data depends not only on whether sequences can be generated, but whether they are biologically plausible, computationally useful, reproducible, and responsibly governed. This Perspective argues that agentic AI can provide the missing orchestration layer for trustworthy synthetic microbial genomics. Rather than treating synthetic data generation as a single model output, agentic workflows can coordinate specialised roles for sequence generation, biological plausibility assessment, taxonomic validation, functional annotation, contamination detection, downstream benchmarking, provenance logging, and governance review. I propose a validation-first agentic framework in which synthetic microbial genomes, plasmids, phages, and metagenomic profiles are iteratively generated, evaluated, revised, and documented before release or downstream use. Such a framework can help transform synthetic microbial genomic data from computational artefacts into auditable scientific infrastructure with explicit validation gates, escalation criteria, and machine-readable provenance.

## 1 Introduction

Microbial genomics is entering a period in which artificial intelligence is no longer used only for classification, annotation, or prediction, but increasingly for representation learning and sequence generation. Genomic foundation models such as Evo, megaDNA, Nucleotide Transformer, GROVER, DNABERT 2, and HyenaDNA show that DNA sequence modelling can learn patterns across long-contexts, diverse species, and complex biological tasks (Nguyen et al., 2024; Shao and Yan, 2024; Dalla Torre et al., 2025; Sanabria et al., 2024; Zhou et al., 2024; Nguyen et al., 2023). This development is especially relevant to microbial genomics, where genomes, plasmids, bacteriophages, mobile genetic elements, and metagenomic communities generate enormous sequence diversity that is difficult to capture through conventional reference-dependent approaches.

Synthetic microbial genomic data can support benchmark construction, rare taxon simulation, metagenomic workflow evaluation, antimicrobial resistance prediction, and controlled ground truth datasets. They may also help address the underrepresentation of microbial lineages, environmental niches, and rare genomic configurations in public databases. However, synthetic microbial genomic data are not automatically trustworthy. A generated genome may appear statistically realistic while containing implausible gene organisation, unstable taxonomic signals, artefactual k-mer profiles, inconsistent functional annotations, or problematic similarity to training data. In microbial genomics, these errors are not trivial. They may distort downstream biological interpretation, weaken benchmarking, or introduce governance concerns when sequences relate to virulence, antimicrobial resistance, pathogenicity, or mobile genetic transfer.

This Perspective argues that the next stage of synthetic microbial genomics should not be defined by more powerful generators alone. It should be defined by agentic workflows that coordinate generation, validation, benchmarking, documentation, and governance. Agentic AI refers to systems capable of planning, tool use, iterative decision making, task decomposition, and coordination across multiple specialised modules or agents (Abou Ali et al., 2025). In the context of synthetic microbial genomics, agentic AI should not replace expert judgement. Rather, it should make validation more systematic, scalable, transparent, and auditable. The core perspective here is not a new generative model, but a role-based agentic validation architecture for synthetic microbial genomic data. Unlike a conventional modular validation pipeline, the proposed system is agentic because each agent can interpret the task objective, select and invoke appropriate tools, evaluate intermediate evidence, and modify the workflow through feedback. Failed or uncertain candidates are returned to earlier agents, rejected, or escalated under predefined human oversight rules, while the provenance agent records the resulting decision path.

## 2 The limitation of generation alone

Recent advances in genomic foundation models have altered expectations about what AI systems can do with biological sequences. Evo was trained on millions of prokaryotic and phage genomes and demonstrated sequence modelling and design from molecular to genome scale (Nguyen et al., 2024). megaDNA introduced a long-context generative model for bacteriophage genomes, illustrating how genome scale structure can be modelled beyond short sequence fragments (Shao and Yan, 2024). Nucleotide Transformer and GROVER show that DNA language models can learn transferable representations and sequence context across large biological corpora (Dalla Torre et al., 2025; Sanabria et al., 2024). These advances make synthetic microbial genomic data increasingly plausible as a computational object. Within this landscape, Evo and megaDNA are most directly aligned with long-context microbial or phage scale generation, whereas Nucleotide Transformer, GROVER, DNABERT 2, and HyenaDNA primarily demonstrate transferable sequence representation, contextual encoding, and long-range genomic modelling that can support validation as well as generation. These models also differ in training data, context length, and primary function, and none independently provides the biological, taxonomic, functional, provenance, and governance checks integrated by the proposed workflow.

Yet plausibility is not the same as validity. Synthetic microbial genomic data must satisfy biological, computational, and governance criteria. A sequence may match broad compositional properties while failing at finer levels of taxonomic coherence, operon organisation, gene density, codon usage, plasmid architecture, phage lifecycle plausibility, antimicrobial resistance context, or ecological compatibility. Likewise, a synthetic metagenomic profile may reproduce community diversity metrics while failing to represent realistic strain variation, abundance distributions, horizontal gene transfer patterns, or functional pathway structure.

The problem is therefore not simply how to generate more synthetic data. The problem is how to produce synthetic microbial genomic data that are suitable for scientific use. Recent benchmarking studies of DNA foundation models show that model performance varies across species, tasks, sequence lengths, pooling strategies, and downstream objectives (Feng et al., 2025). This reinforces a central point for synthetic microbial genomics: validation must be task aware. A synthetic genome suitable for testing taxonomic classification may not be suitable for benchmarking antimicrobial resistance prediction. A synthetic phage genome useful for long-context sequence modelling may not be appropriate for functional inference. A synthetic metagenomic community useful for abundance profiling may fail for assembly or binning evaluation. This is consistent with recent microbiome and metagenomic machine learning literature, which emphasises that microbial community analysis depends on complex interactions among sequencing modality, taxonomic classification, functional profiling, benchmark design, and pipeline specific assumptions (Yan et al., 2025; Przymus et al., 2025; Pope et al., 2025; Meyer et al., 2022).

## 3 Agentic AI as an orchestration layer

An agentic workflow for synthetic microbial genomics should begin with a clear scientific objective. The objective may be to benchmark a taxonomic classifier, simulate rare environmental taxa, evaluate a metagenomic assembler, test plasmid detection tools, generate negative controls, or construct privacy preserving training data. Once the objective is defined, the agentic system decomposes the task into specialised roles. Each agent has a bounded function, defined inputs, explicit outputs, and validation responsibility.

**FIGURE 1:**
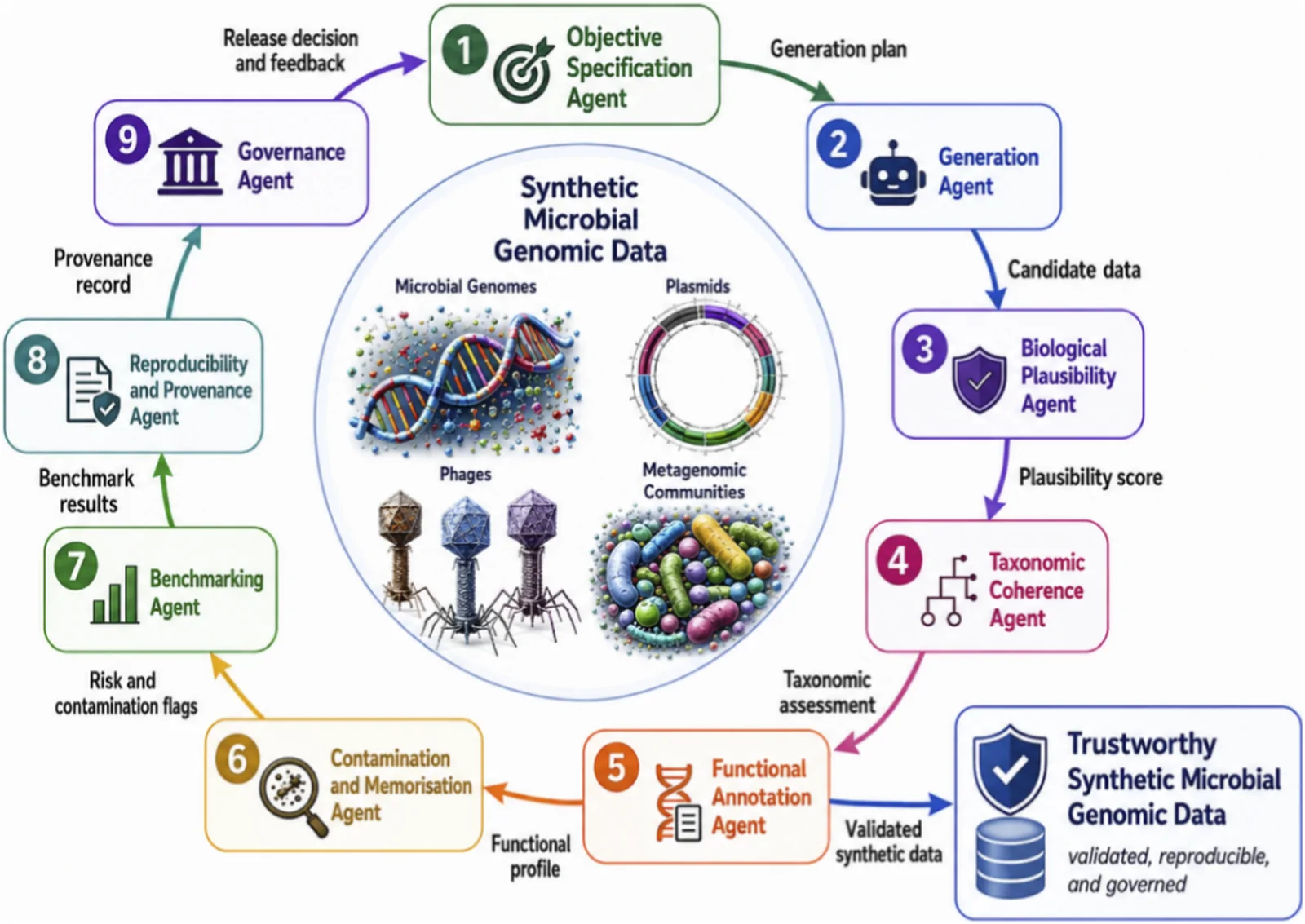
Agentic AI workflow for trustworthy synthetic microbial genomics.

As seen from Figure 1, the first role is the objective specification agent. This agent translates the research goal into measurable requirements. For example, if the objective is to benchmark metagenomic taxonomic classification, it defines target taxa, abundance distributions, read depth, sequencing modality, strain variation, and expected ground truth. If the objective is plasmid simulation, it defines plasmid length ranges, replication features, host range assumptions, gene content constraints, and acceptable similarity thresholds to known plasmids.

The generation agent uses an appropriate generative model or simulation method to produce candidate microbial genomes, plasmids, phages, or metagenomic profiles. Its task is not to declare success, but to produce initial candidates for validation. It should attach generation metadata, including model version, sampling settings, prompts if applicable, reference constraints, random seeds, and excluded sequence classes.

The third role is the biological plausibility agent that checks whether generated sequences satisfy fundamental biological expectations. It evaluates genome length, GC content, coding density, k-mer distributions, codon usage, open reading frame organisation, repetitive elements, structural irregularities, and taxon specific compositional signatures. For plasmids and phages, it also examines architecture specific features such as replication related genes, capsid or tail associated regions, lifecycle markers, mobility signatures, and host compatibility indicators. Operationally, this agent may combine compositional checks with genome quality, annotation, and mobile element assessment using tools such as CheckM2, Bakta, MOB suite, geNomad, and VirSorter2, while reporting deviations from the target distribution rather than relying on a single plausibility score (Robertson and Nash, 2018; Schwengers et al., 2021; Guo et al., 2021; Chklovski et al., 2023; Camargo et al., 2024).

The taxonomic coherence agent asks whether the synthetic sequence has a stable and interpretable taxonomic identity. It compares multiple taxonomic classification tools, for example, GTDB Tk for bacterial and archaeal genomes together with an independent classifier where appropriate, checks whether assignments remain consistent across databases and taxonomic ranks, and flags ambiguous or contradictory outputs for regeneration or expert review (Chaumeil et al., 2020). In metagenomic datasets, it assesses whether synthetic community composition reflects the intended ecological or experimental scenario. This is crucial because microbial sequence space is shaped by strain variation, horizontal gene transfer, and incomplete reference databases.

The fifth role is the functional annotation agent that evaluates predicted genes, pathways, antimicrobial resistance markers, virulence associated regions, metabolic functions, and mobile genetic elements. The objective is not merely to annotate the sequence, but to determine whether annotations are internally coherent. For example, a generated bacterial genome should not contain arbitrary combinations of genes that are compositionally plausible but biologically implausible. For plasmids, functional coherence should require compatible replication, mobilisation or conjugation signals, accessory genes, host range assumptions, and genomic neighbourhoods; for phages, it should require coherent capsid, tail, terminase, packaging, lysis, lysogeny, and host compatibility signals.

The contamination and memorisation agent evaluates whether generated sequences show excessive similarity to training data, reference genomes, known pathogenic regions, or restricted sequence classes. It also checks for chimeric artefacts, duplicated segments, unnatural sequence joins, and high-risk motifs, defined here as regions or translated products with concerning similarity to regulated pathogen associated genes, toxin or virulence domains, antimicrobial resistance determinants in mobile contexts, or other sequence classes defined by institutional, national, or synthesis screening policies. Detection should combine nucleotide similarity search, translated sequence comparison, domain-based screening, sequence of concern screening, and expert review rather than unrestricted language model judgement. This role is central to both scientific validity and governance, especially because synthetic nucleic acid oversight is increasingly moving toward sequence-level screening and AI aware risk mitigation.

The seventh role is the benchmarking agent that runs the synthetic data through downstream microbial genomics pipelines. These may include assembly, binning, taxonomic classification, functional annotation, antimicrobial resistance detection, plasmid reconstruction, phage identification, or metagenomic profiling. The purpose is to determine whether synthetic data behave as intended under realistic computational use. This step converts validation from a static sequence-level judgement into a task-specific evaluation. For this reason, downstream validation should include taxonomic classification, long read metagenomic classification, and pangenome aware evaluation where appropriate, since recent studies show that classifier performance, mock community benchmarking, and microbial pangenome resources remain central to reproducible microbial genomics (Van Uffelen et al., 2024; Marić et al., 2024; Sun et al., 2025).

The reproducibility and provenance agent records the entire workflow, including input objectives, model configurations, reference databases, software versions, decision rules, rejected candidates, validation scores, expert interventions, and release conditions. The record should be exportable in a machine-readable format, for example, JSON LD through RO Crate or a W3C PROV compatible structure, so that synthetic datasets can be audited beyond narrative reporting (Lebo et al., 2013; Soiland Reyes et al., 2022). Without this role, synthetic microbial genomic data may become difficult to interpret, reproduce, or audit.

Finally, the ninth role is the governance agent. This agent evaluates whether data should be released openly, shared under controlled conditions, modified, or withheld. It incorporates criteria from synthetic nucleic acid screening, dual use governance, biosafety considerations, and institutional policy. NIST and OECD guidance on AI enabled biological design and synthetic biology governance emphasise that technical innovation must be accompanied by risk assessment, human oversight, and biosecurity aware controls (NIST, 2024; OECD, 2025). Governance therefore becomes part of the workflow rather than an afterthought.

Figure 1 summarises the proposed agentic AI workflow from objective specification to governed release, while Table 1 translates the same framework into operational validation gates, pass conditions, and failure responses for trustworthy synthetic microbial genomics.

**TABLE 1:** Validation gates for trustworthy synthetic microbial genomics.

Workflow gate	Validation question and operational checks	Pass or escalation condition	Failure response
Objective specification	What is the synthetic dataset intended to test or simulate? Target organism or community, data type, benchmark task, intended use, exclusion criteria, success metric	Objective, target taxa, data type, benchmark task, and release context are explicit	Redefine scope before generation
Synthetic generation	Are candidate genomes, plasmids, phages, or communities produced with sufficient metadata? Model, simulation method, sampling settings, random seed, reference constraints, excluded sequence classes	Candidate data include complete generation metadata and reproducible settings	Regenerate with documented settings
Biological plausibility	Do the outputs resemble realistic microbial genomic entities? Length, GC content, coding density, ORF structure, codon use, k-mer distribution, genome quality, plasmid or phage architecture	Values remain within the target distribution and no major structural inconsistency is detected	Reject, revise, or return to generation
Taxonomic coherence	Is microbial identity stable across tools and databases? GTDB Tk or comparable taxonomic assignment, independent classifier agreement, rank stability, database consistency	Assignment is stable across tools at the intended rank; conflict at genus, species, or host level triggers expert review	Flag ambiguity, regenerate, or restrict use
Functional coherence	Are genes, pathways, AMR markers, and mobile elements biologically consistent? Gene annotation, pathway structure, AMR markers, virulence associated regions, mobile genetic elements, plasmid and phage modules	Functional profile is biologically coherent for the intended organism, plasmid, phage, or community	Remove, revise, reject, or escalate
Contamination and safety	Are there copied, chimeric, restricted, or sensitive sequence elements? Similarity screening, chimeric joins, copied regions, sequence of concern screening, high risk motif detection	No excessive reference similarity, restricted sequence class, unresolved safety hit, or sensitive mobile context is detected	Escalate to expert review, degrade, restrict, or withhold
Downstream benchmarking	Does the dataset perform as intended in microbial genomics pipelines? Assembly, binning, taxonomic classification, plasmid reconstruction, phage identification, AMR prediction, functional profiling	Synthetic data behave as intended for the specified benchmark task and do not create misleading outputs	Recalibrate dataset, revise objective, or regenerate
Provenance and auditability	Can the workflow be reproduced and reviewed? Provenance and auditability Operational checks: Tools, databases, model versions, parameters, thresholds, rejected candidates, expert decisions, release status	Complete human readable and machine-readable provenance record is available	Do not release until documentation is complete
Governance review	Can the data be safely shared? Release category, intended user, sequence screening result, sensitive annotation, institutional policy, controlled access requirement	Open release, controlled access, degraded release, or withholding decision is justified	Restrict, degrade, withhold, or refer to institutional oversight

As seen from Figure 1 and Table 1, the agents interact iteratively by passing each synthetic candidate through generation, biological validation, taxonomic assessment, functional annotation, contamination screening, benchmarking, provenance logging, and governance review. Any failure, ambiguity, or risk flag returns the candidate to the relevant earlier agent for refinement, rejection, or expert escalation before release. At implementation level, each agent receives the candidate data (x), the objective specification (O), and the accumulated provenance state (P), and returns validation evidence, a task-specific score, a confidence or risk flag, and one of four actions: pass, revise, reject, or escalate. Scores may include normalized GC content deviation, k-mer distribution distance, ORF density, taxonomic classifier agreement, functional annotation consistency, and sequence similarity risk. Thresholds should be predefined from reference distributions or benchmark specific tolerances rather than treated as universal constants. A passing result advances the candidate to the next mandatory gate. A revision result returns the candidate and its failure evidence to the relevant upstream agent, rejection terminates processing, and escalation invokes expert review. Release occurs only when all mandatory validation gates pass and the Governance Agent authorizes open or controlled release.

## 4 Illustrative validation workflow

To illustrate the workflow without converting this Perspective into an experimental study, consider a candidate synthetic plasmid generated for benchmarking plasmid detection and annotation tools in Enterobacterales. The Objective Specification Agent first defines the intended host context, plasmid length range, benchmark task, expected gene categories, similarity limits, and release conditions. The Generation Agent then produces a candidate sequence with model settings, sampling parameters, constraints, random seed, and excluded sequence classes.

The Biological Plausibility Agent evaluates length, GC content, coding density, open reading frame structure, codon use, k-mer pattern, and plasmid architecture. The Taxonomic Coherence Agent checks whether host or taxonomic signals remain stable across independent classifiers. The Functional Annotation Agent then evaluates whether replication, mobilisation, conjugation, resistance, virulence associated, and accessory genes form a coherent biological profile rather than an arbitrary collection of plausible annotations. The Contamination and Memorisation Agent screens for excessive reference similarity, chimeric joins, copied regions, and high-risk motifs. The Benchmarking Agent tests whether downstream tools recover the intended plasmid identity and annotation profile.

If any agent detects low confidence taxonomy, inconsistent gene neighbourhoods, unresolved safety hits, or incomplete provenance, the candidate is returned for refinement or escalated to expert review. The same logic applies to synthetic metagenomic profiles, where validation should also consider richness, abundance distribution, strain variation, niche consistency, co-occurrence structure, and functional pathway plausibility. For example, a plasmid that satisfies compositional criteria but lacks coherent replication or mobility modules would be returned for revision or rejected. This illustrative scenario clarifies the proposed workflow logic rather than reporting empirical performance. Future work should compare an implemented agentic workflow with a fixed modular pipeline using gate failure rates, false acceptance, expert escalation frequency, runtime, and reproducibility.

## 5 Governance and responsible release

Synthetic microbial genomic data intersect with sensitive domains. Sequences may relate to antimicrobial resistance, virulence, pathogenicity, host adaptation, environmental release, or synthetic construct design. Recent work on generative AI in microbial evolution, synthetic nucleic acid oversight, and AI assisted biological design argues that governance must address explainability, biosafety, implementation gaps, screening capacity, and the changing risks introduced by increasingly automated biological systems (Sufi, 2025; Gillum, 2025; Berman, 2026). Recent inter tool evaluation of synthetic nucleic acid screening further indicates that governance depends not only on policy principles but also on measurable screening performance across tools and datasets (Laird et al., 2025). Therefore, an agentic workflow must include release decision rules. Operationally, the Governance Agent should incorporate sequence of concern screening, customer or user legitimacy considerations where relevant, and documented comparison with institutional policies or recognized synthesis screening practices.

A simple governance model may classify synthetic outputs into three release categories. Low risk outputs may be released openly with full provenance and validation reports. Moderate risk outputs may be shared under controlled access, with restrictions on sequence resolution, sensitive annotations, or downstream synthesis use. High risk outputs may be withheld, degraded, or restricted to secure review. The governance agent should not make these decisions alone. Expert escalation should be triggered by unresolved sequence of concern matches, conflicting taxonomic assignment, functionally inconsistent plasmid or phage architecture, high similarity to restricted or pathogenic references, uncertain intended use, or incomplete provenance. It should flag cases for expert review and institutional oversight.

Human expertise remains essential. Agentic AI can coordinate validation tasks and reduce manual burden, but it cannot fully determine biological meaning, biosafety acceptability, or social legitimacy. The appropriate model is therefore human-supervised autonomy, where agents perform structured checks and experts retain authority over final release decisions. This position is also consistent with broader agentic AI scholarship, which frames agentic systems as decision support and orchestration mechanisms rather than replacements for domain expertise (Hosseini and Seilani, 2025).

## 6 Discussion

This Perspective reframes synthetic microbial genomics as a validation and governance problem, rather than merely a sequence generation problem. Synthetic microbial genomes, plasmids, phages, and metagenomic communities must be biologically plausible, computationally useful, reproducible, and responsibly governed before downstream use or release. Agentic AI offers a practical route by coordinating generation, validation, benchmarking, provenance recording, and governance within a single iterative workflow. URL2Graph++ similarly shows that safety relevant AI can benefit from multi signal orchestration, combining semantic, structural, character level, graph based, and gated fusion signals rather than relying on a single classifier output (Tian et al., 2026). Broader biomedical AI developments similarly demonstrate the value of integrated and validation aware systems, including heterogeneous information network models for biomolecular interaction prediction and drug repositioning, diffusion-based protein peptide docking, quantum grounded virtual discovery, and interactive repositioning platforms (Zhao et al., 2022; 2024; 2025; 2026; Su et al., 2026). These advances reinforce the need for agentic genomic workflows that combine structured knowledge, tool orchestration, task-specific validation, and auditable decision making.

The field now needs clearer reporting standards for synthetic microbial genomic data, including model identity, generation settings, reference databases, validation criteria, contamination checks, similarity screening, expert review procedures, release conditions and machine-readable provenance records. Journals and repositories should require provenance statements when synthetic datasets involve microbial genomes, plasmids, phages, antimicrobial resistance, pathogenicity related features, or other sensitive biological content.

Future benchmark suites should test whether synthetic data preserve taxonomic realism, functional coherence, ecological plausibility, and downstream pipeline utility. The objective is not to replace microbial genomics expertise, but to make expert guided validation more scalable, systematic, and auditable. The next Frontier should therefore be validation-first synthetic microbial genomics, where agentic AI transforms synthetic data generation from an isolated computational act into a governed scientific process.

## Data Availability

The original contributions presented in the study are included in the article/supplementary material, further inquiries can be directed to the corresponding author.
